# Ameliorating role of microRNA-378 carried by umbilical cord mesenchymal stem cells-released extracellular vesicles in mesangial proliferative glomerulonephritis

**DOI:** 10.1186/s12964-022-00835-1

**Published:** 2022-03-09

**Authors:** Wenbiao Chen, Feng Zhang, Xianliang Hou, Huixuan Xu, Donge Tang

**Affiliations:** 1 Central Laboratory, People’s Hospital of Longhua, The Affiliated Hospital of Southern Medical University, Jianshe East Road, Longhua District, Shenzhen, 518109 Guangdong People’s Republic of China; 2 Department of Respiratory Medicine, People’s Hospital of Longhua, The Affiliated Hospital of Southern Medical University, Shenzhen, 518109 Guangdong People’s Republic of China; 3 Clinical Medical Research Center, Guangdong Provincial Engineering Research Center of Autoimmune Disease Precision Medicine, The First Affiliated Hospital of Southern University of Science and Technology, The Second Clinical Medical College of Jinan University, Shenzhen People’s Hospital, No. 1017 Dongmen North Road, Shenzhen, 518020 Guangdong People’s Republic of China; 4 Intensive Care Unit, The First Affiliated Hospital of Jinan University, Guangzhou, 510632 Guangdong People’s Republic of China

**Keywords:** Umbilical cord mesenchymal stem cells, Extracellular vesicles, Mesangial proliferative glomerulonephritis, microRNA-378, PSMD14, TGF-β1/Smad2/3 signaling pathway

## Abstract

**Background:**

Mesenchymal stem cells (MSCs) and their released extracellular vesicles (Evs) have shown protective effects against kidney diseases. This study aims to study the functions of umbilical cord MSCs-released Evs (ucMSC-Evs) and their implicated molecules in mesangial proliferative glomerulonephritis (MsPGN).

**Methods:**

A rat model of MsPGN was induced by anti-Thy-1.1, and rat mesangial cells (rMCs) HBZY-1 were treated with PDGF-BB/DD to mimic MsPGN condition in vitro. Rats and cells were treated with different doses of ucMSC-Evs, and then the pathological changes in renal tissues and proliferation of rMCs were determined. Differentially expressed microRNAs (miRNAs) after Evs treatment were screened by microarray analysis. The interactions among miR-378, PSMD14, and TGFBR1 were analyzed. Gain- and loss-of function studies of miR-378 and PSMD14 were performed to explore their effects on tissue hyperplasia and rMC proliferation and their interactions with the TGF-β1/Smad2/3 signaling pathway.

**Results:**

The ucMSC-Evs treatment ameliorated mesangial hyperplasia and fibrosis in rat renal tissues and suppressed the aberrant proliferation of rMCs in a dose-dependent manner. miR-378 was the most upregulated miRNA in tissues and cells after ucMSC-Evs treatment. miR-378 directly targeted PSMD14, and PSMD14 maintained the stability of TGFBR1 through deubiquitination modification, which led to TGF-β1/Smad2/3 activation. Either miR-378 knockdown or PSMD14 overexpression diminished the protective functions of ucMSC-Evs by activating the TGF-β1/Smad2/3 signaling pathway.

**Conclusion:**

UcMSC-Evs ameliorate pathological process in MsPGN through the delivery of miR-378, which suppresses PSMD14-mediated TGFBR1 stability and inactivates the TGF-β1/Smad2/3 signaling pathway to reduce tissue hyperplasia and rMC proliferation.

**Graphical Abstract:**

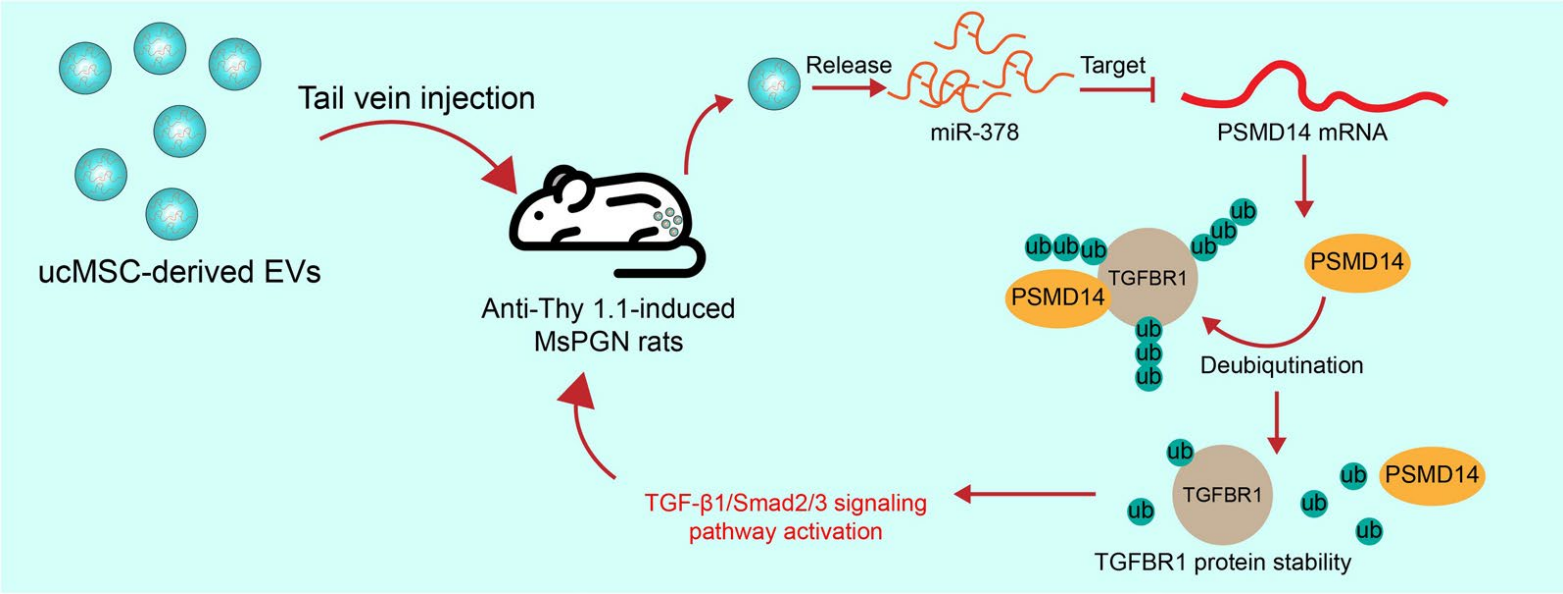

**Video abstract**

**Supplementary Information:**

The online version contains supplementary material available at 10.1186/s12964-022-00835-1.

## Background

Mesangial proliferative glomerulonephritis (MsPGN) is characterized by the diffuse proliferation of mesangial cells (MCs) and deposition of mesangial matrix, which contributes to renal interstitial fibrosis, irreversible progressive glomerulosclerosis, and end-stage renal disease (ESRD) [1, 2]. MsPGN, takes up approximately 60% of all primary GN cases in China, is a predominant cause of chronic kidney disease, chronic renal failure, and uremia [3]. However, there are limited therapeutic options available for MsPGN treatment, and pharmacological interventions inhibiting MC proliferation and matrix accumulation are primary options to retard the GN progression [4].

Mesenchymal stem cells (MSCs) are progenitor multipotent cells abundantly existed in umbilical cord (uc) blood, adipose tissue, and bone marrow many tissues, which serve as an ideal candidate with therapeutic potentials owing to their secretory capacity, mainly including extracellular vesicles (Evs) [5]. MSCs participate in the repair of tissues, especially kidney, primarily by the release of Evs and their cargoes including lipids, microRNAs (miRNAs), mRNAs, and proteins [6, 7]. The functions of ucMSCs-derived Evs (ucMSC-Evs) in MsPGN are not fully explained. miRNAs are a major class of cargoes of Evs that maintain the normal function of human body conditions through the multipotent regulation in cell migration, proliferation, differentiation, and apoptosis [8]. However, aberrant expression of miRNAs is frequently correlated with many diseases including chronic kidney diseases [8]. Studies have reported that miRNAs can influence proliferation of glomerular MCs and accumulation of extracellular matrix (ECM) [9, 10]. In this study, miR-378 was screened as the most upregulated miRNA after administration of ucMSCs and therefore selected as the study subject.

The transforming growth factor-β1 (TGF-β1)/mothers against decapentaplegic homolog (Smad) signaling pathway plays a crucial role in the prolonged glomerulosclerosis and the progression of chronic kidney diseases [11]. After binding to the TGF-β receptors (TGFBRs), TGF-β1 activates two critical downstream mediators, Smad2 and Smad3, to fulfill its functions including ECM production [12]. Interestingly, miR-378 has been reported to suppress mesangial hypertrophy, expression of collagens and α-smooth muscle actin (α-SMA) (biomarkers of ECM) in mice increased by TGF-β1 and Smad3 [13]. miRNAs are well known to govern gene expression to exert their functions. In the study, we found an enrichment of ubiquitinated protein degradation-related pathways by the predicted target mRNAs of miR-378. Among the miR-378 targets, proteasome 26S subunit, non-ATPase 14 (PSMD14), a deubiquitinating enzyme that has been found to trigger the deubiquitination to maintain the stability of TGFBRs [14], attracted our attention. We hypothesized that miR-378 carried by the Evs possibly suppresses PSMD14 expression to modulate the activity of TGFBRs and the following TGF-β1/Smad2/3 signaling pathway, therefore controlling MC proliferation and MsPGN progression.

The Thy-1.1 antibody is commonly used for MsPGN induction in animal models [15], while platelet-derived growth factors (PDGF-A, -B, -C and -D) are the best-known growth factors participating in MC proliferation [16–18]. In the study, we induced a rat model with MsPGN by anti-Thy-1.1, and treated rat MCs (rMCs) with PDGF-BB/DD, which were treated with ucMSC-derived Evs to explore their functions in MsPGN progression.

## Materials and methods

### Ethics statement

The animal experimental protocol was approved by the Committee on the Ethics of Animal Experiments of People's Hospital of Longhua. All animal procedures were performed according to the Guide for the Care and Use of Laboratory Animals published by the National Institutes of Health (NIH, Bethesda, Maryland, USA). Great efforts were made to reduce the pain in animals.

### Animals, reagents, antibodies and primers

Sprague–Dawley rats (180–200 g) were purchased from the Shanghai Lab. Animal Research Center. HEK-293 T cells were acquired from American Type Culture Collection (ATCC, Manassas, VA, USA). The rabbit polyclonal anti-Thy-1.1 was prepared as previously reported [19]. The miRNA microarray product was from Agilent Technologies (Palo Alto, CA, USA). Lipofectamine 2000 used for transfection was purchased from Invitrogen, Thermo Fisher Scientific Inc (Waltham, MA, USA). The pGL3 promoter reporter vectors, and the dual-luciferase reporter assay kit were provided by Promega Corp. (Madison, WI, USA). A PrimeScript™ RT kit was purchased from (TaKaRa, Holdings Inc., Kyoto, Japan). The Bulge Loop™ RT primers and primers used for reverse transcription quantitative polymerase chain reaction (RT-qPCR) were designed and synthetized by RiboBio Co., Ltd. (Guangdong, China). SYBR®Green Master Mix and HiScript®IIqRT SuperMix were procured from Vazyme Biotech Co., Ltd. (Nanjing, Jiangsu, China). The antibodies against glyceraldehyde-3-phosphate dehydrogenase (GAPDH, #60004-1-Ig), TGFB1 (#21898-1-AP, antibody specificity determined by KO validation), proliferating cell nuclear antigen (PCNA, #10205-2-AP, antibody specificity determined by KO validation). and PUMA (#55120-1-AP, antibody specificity determined by orthogonal validation) were purchased from Protein tech Group, Inc. (Wuhan, Hubei, China). Antibody against phospho-SMAD2 (Ser465/Ser467) (#MA5-15122, antibody specificity determined by cell treatment validation) was purchased from Thermo Fisher Scientific. Antibodies against phospho-SMAD3 (Ser423/Ser425) (#GTX00969, antibody specificity determined by Orthogonal validation), ɑ-SMA (#GTX100034), Cyclin D2 (#GTX32545), and Bcl-2 associated X (Bax, #GTX32465, antibody specificity determined by KO validation) were purchased from Genetex Inc. (San Antonio, TX, USA). Horseradish peroxidase (HRP)-conjugated anti-mouse or anti-rabbit immunoglobulin G (IgG) was purchased from Cell Signaling Technology (Beverly, MA, USA). The Annexin V cell apoptosis detection kit was acquired from Becton Dickinson (BD) Biosciences (San Jose, CA, USA). PDGF-BB and PDGF-DD were provided by R&D Systems (Minneapolis, MN, USA).

### Culture and identification of the umbilical cord mesenchymal stem cells (ucMSCs)

A uc-MSC line (CP-R302) was acquired from Procell Life Science & Technology Co., Ltd. (Wuhan, Hubei, China). The ucMSCs were cultured in Dulbecco's modified Eagle's medium (DMEM) supplemented with 10% fetal bovine serum (FBS), 100 U/mL penicillin and 100% μg/mL streptomycin (Solarbio Science & Technology Co., Ltd., Beijing, China) at 37 °C with 5% CO_2_. The adipogenic differentiation and osteogenic differentiation abilities of uc-MSCs were determined using the adipogenic and osteogenic induction kits (Cyagen Biosciences Inc., Guangzhou, Guangdong, China). Correspondingly, Oil red O staining and Alizarin red staining were performed according to the kit’s instructions.

### Extraction and identification of the Evs

Upon reaching an 80% confluence, the ucMSCs were cultured in an Evs-exhausted FBS-supplemented DMEM under normal oxygen condition for 48 h. Next, the medium was collected and centrifuged at 300×g for 10 min, and then at 2000×g at 4℃ for 10 min. After that, a 0.22-μm sterile filter (Steritop™ Millipore, Billerica, MA, USA) was used to filter the supernatant from the cells and cell debris. After that, the supernatant was further centrifuged at 4000× in an Amicon Ultra-15 centrifugal filter (Millipore), and the hyper-filtered supernatant was collected. To purify the Evs, the collected supernatant was loaded on a sterile Ultra-Clear™ tube (Beckman Coulter, Asphalt, CA, USA) and ultra-centrifuged at 4 °C for 10 min. The precipitates containing the ucMSC-Evs were collected using an 18-G probe, diluted in phosphate-buffered saline (PBS), and centrifuged at 4000×g at 4 °C to a final volume of 200 μL. The final concentration of the stored Evs was 200 pg/μL protein.

A Nanosight LM10 System (Malvern Panalytical, Malvern, Worcs, UK) was used to analyze the diameter distribution of the collected particles. The shape of the particles was observed under a transmission electron microscope (TEM, Tecnai 12, Philips, Best, The Netherlands). The specific biomarkers of Evs including TSG101 (ab125011, Abcam Inc., Cambridge, MA, USA), CD9 (ab92726, Abcam), CD63 (ab193349, Abcam), and CD81 (ab109201, Abcam) were examined by western blot analysis. A bicinchoninic acid (BCA) kit (Thermo Fisher Scientific) was used to determine protein concentration. The optical density (OD) value was read by a microplate reader (ELx800, Bio-Tek Instruments Inc., Winooski, VT, USA) at 562 nm.

### MsPGN in rats induced by anti-Thy-1.1

The SD rats were allocated into Thy-1.1 group (n = 42) and sham group (n = 6). Rats in the Thy-1.1 group were administrated with rabbit serum containing anti-Thy-1.1 through an intravenous injection [20–22], while rats in the sham group were given an equal volume of anti-Thy-1-deprived rabbit serum.

To investigate the effects of ucMSC-Evs on the anti-Thy-1.1-induced MsPGN in rats, the model rats (n = 42) were allocated into seven groups: MsPGN group (model rats without any other treatment), MsPGN + PBS group (model rats treated with PBS), MsPGN + 20 μg/mL/kg Evs group (model rats were administrated with 20 μg/mL/kg ucMSC-Evs), MsPGN + 40 μg/mL/kg Evs group (model rats were administrated with 40 μg/mL/kg ucMSC-Evs), Evs + sodium dodecyl sulfate (SDS) group (model rats were administrated with 40 μg/mL/kg SDS-treated ucMSC-Evs), Evs/Inhibitor NC group (model rats were administrated with 40 μg/mL/kg ucMSC-Evs/Inhibitor NC) and Evs/miR-378 inhibitor group (model rats were administrated with 40 μg/mL/kg ucMSC-Evs/miR-378 Inhibitor). There were 6 rats in each group. Injection was performed three times per week for a total of two weeks, after which the animals were euthanized via an intraperitoneal injection of pentobarbital sodium (150 mg/kg), and the tissues of renal cortex were collected. Animal death but not a cardiac arrest was confirmed by the limb stiffness, the losses of blink reflex, nerve reflex and heartbeat. Thereafter, the rat renal tissues were collected for the subsequent experiments, and the left ones were used for RNA and protein measurement, and the right ones were used for histological examinations.

### Detection of proteinuria, serum creatinine and cystatin-C (Cys-C) expression rats

On the 14^th^ days (d) after Evs or PBS treatment, the urine samples (24-h) were collected. The concentration of urinary protein was determined using a Bradford Protein Assay Kit (Solarbio). The rat blood samples were collected, allowed to stand at 37 °C for 25 min and centrifuged 1006.2×g for 20 min to collect the supernatant, which was equally divided into 200 µL and stored at − 80 °C. The creatinine and Cys-C levels in serum samples were determined using enzyme-linked immunosorbent assay (ELISA) kits of serum creatinine (catalog #: K625, BioVision, Milpitas, CA, USA) and Cys-C kit (Catalog #: E4305, Biovision), respectively. The OD value at 450 nm was determined using the microplate reader.

### Renal morphology and immunohistochemical (IHC) staining

The rat renal tissues were fixed in 10% formalin, embedded in paraffin, cut into 4-μm sections, and dewaxed for periodic acid schiff (PAS) staining (Solarbio). The histopathological characteristics of the glomerulus in tissue sections were observed under a microscope (BX5, Olympus, Tokyo, Japan). The number of total PAS-positive cells in each glomerular cross section (150 sections in each group) was calculated using a CellSens Standard digital imaging software (Olympus). The hematoxylin–eosin (HE) staining was further performed. In brief, the tissue sections were stained by hematoxylin and eosin (Sigma-Aldrich Chemical Company, St Louis, MO, USA) for 3 min, and successively soaked in 95% alcohol, absolute ethanol I and absolute ethanol II for 3 min, and then in xylene I and xylene II for 5 min, and sealed by neutral resin (Solarbio) for microscopy observation. The damage in renal tubules was evaluated, and the inflammatory response was scored (0–4): 0, normal kidney; (1) minimum necrosis (< 5%); (2) mild necrosis (5–25%); (3) moderate necrosis (25–75%), and (4) severe necrosis (> 75%). In addition, Masson's trichrome staining (Sigma-Aldrich) was performed to examine the fibrosis in renal tissues according to the manufacturer’s instructions. The normal tissues were stained in red, whereas the fibrotic tissues were stained in blue.

IHC staining was performed as previously described [23]. In brief, the paraffin-embedded sections were dewaxed. After antigen retrieval, the sections were treated with H_2_O_2_ to block the activity of the endogenous peroxidase. After that, the sections were incubated with the primary antibodies at 37 °C for 45 min, and then incubated with 100 μL Reagent A (HRP-labeled ChemMate Envision reagent) from a common anti-rabbit or anti-rat IHC staining kit (Envision Detection Kit, GK500705, Dako, Agilent). Next, 3,3′-diaminobenzidine (Sigma-Aldrich) was used for color development. The sections were counter-stained with hematoxylin, and the positively-stained cells (brown) were observed and captured under the microscope.

### Culture of rMCs

A rMC cell line HBZY-1 (CL-0092) was acquired from Procell as well. Cells were cultured in 10% FBS-DMEM at 37 °C with 5% CO_2_. When the cell confluence reached 75–85%, the cells were treated with PBS (blank) or 100 ng/mL PDGF-BB or PDGF-DD for 24 h. The PDGF-treated cells were further treated with different doses of ucMSC-Evs (0, 20, and 40 μg/mL, respectively).

### Determination of the viability of rMCs

The rMCs were seeded in 96-well plates (Corning Inc., Corning, NY, USA) at 5 × 10^3^ cells per well. Cell viability was determined using a CellTiter-Glo® (CTG) detection kit (Promega) in line with the kit’s instructions. The proliferative activity of cells was determined by a 5-ethynyl-2′-deoxyuridine (EdU) labeling kit (Solarbio). The proliferating cells were stained by EdU, whereas the nuclei of all cells were stained by 4′, 6-diamidino-2-phenylindole (DAPI, Beyotime, Biotechnology Co. Ltd., Shanghai, China). The labeling images were captured by a laser confocal microscope (FV3000, Olympus). In addition, the ratio of live to dead cells was confirmed by acridine orange/ethidium bromide (AO/EB) double fluorescence staining. Cells were cultured in a mixture of 20 µL AO and 20 µL EB (both from Solarbio) for 5 min, and the cell morphology was analyzed under a fluorescence microscope (ECLIPSE Ti, Nikon Instruments Inc., Tokyo, Japan).

Apoptosis of cells was further examined using an Annexin V apoptosis detection kit (BD Bioscience). In brief, a total of 10^5^ cells were resuspended in 100 μL 1 × binding buffer. The samples were added with 5 μL Annexin V fluorescein isothiocyanate (FITC) for 15 min, and then added with 5 μL phycoerythrin-labeled propidium iodide. After that, the apoptosis of cells was instantly analyzed by a flow cytometer (BD). A Hoechst 33,258 kit (Solarbio) was used to measure the number of apoptotic cells. In brief, cells were sorted in 24-well plates at 2.5 × 10^4^ cells per well. The cells were fixed with 4% paraformaldehyde for 10 min, washed in PBS, and stained with Hoechst 33,258 (200 μL per well) in the dark for 30 min. The staining was observed under the fluorescence microscope with 5 random fields included.

#### Examination of collagen deposition in cells

Collagen deposition in cells was examined using a Total Collagen kit (Abcam). First, cell lysates were hydrolyzed in an alkaline environment. The supernatant was incubated with oxidation reagent mix at room temperature for 20 min, incubated with developer at 37 °C for 5 min, and then incubated with DMAB solution at 65 °C for 45 min. The OD value at 560 nm was examined.

#### Reverse transcription quantitative polymerase chain reaction (RT-qPCR)

Total RNA from rat renal tissues and rMCs was extracted using the TRIzol Reagent (Invitrogen, Thermo Fisher Scientific) and reverse transcribed into cDNA using a PrimeScript™RT kit (TaKaRa) or HiScript®qRT SuperMix (Vazyme). The cDNA templates were used for real-time qPCR on a LightCycler®480 System (Roche Ltd, Basel, Switzerland) using the SYBR Green Master Mix (Vazyme). The primers are listed in Table 1. GAPDH and U6 were used as the internal controls for mRNAs and miRNA, respectively. Relative gene expression was determined by the 2^−ΔΔCt^ method.

**Table 1:** Primer sequences for RT-qPCR

Gene	Primer sequence (5′–3′)
Cyclin D2	F: GCAGAAGGACATCCAACCGTAC
	R: ACTCCAGCCAAGAAACGGTCCA
PCNA	F: AGTTTTCTGCGAGTGGGGAG
	R: AAGACCTCAGAACACGCTGG
Bax	F: CACGTCTGCGGGGAGTCA
	R: TAGGAAAGGAGGCCATCCCA
Fibronectin-1	F: GGATCCCCTCCCAGAGAAGT
	R: GGGTGTGGAAGGGTAACCAG
Collagen IV	F: CGGGTGTGAAAAGACCTATCGG
	R: CTGGCATTCCTCTGACGCCTTT
PUMA	F: ACCGCTCCACCTGCCGTCAC
	R: ACGGGCGACTCTAAGTGCTGC
PSMD14	F: GTCAGTGTGGAGGCAGTTGATC
	R: CCACACCAGAAAGCCAACAACC
TGFBR1	F: TGCTCCAAACCACAGAGTAGGC
	R: CCCAGAACACTAAGCCCATTGC
GAPDH	F: GTCTCCTCTGACTTCAACAGCG
	R: ACCACCCTGTTGCTGTAGCCAA
miR-212-3p	F: ACCTTGGCTCTAGACTGCTT
	R: GAACATGTCTGCGTATCTC
miR-219a	F: TGATTGTCCAAACGCAATTC
	R: GAACATGTCTGCGTATCTC
miR-455	F: TGTGCCTTTGGACTACATC
	R: GAACATGTCTGCGTATCTC
miR-483-5p	F: ACGGGAGAAGAGAAGGGA
	R: GAACATGTCTGCGTATCTC
miR-511-3p	F: TGCCTTTTGCTCTGCACTC
	R: GAACATGTCTGCGTATCTC
miR-378-5p	F: CTGACTCCAGGTCCTGTG
	R: GAACATGTCTGCGTATCTC
miR-202-3p	F: TTCCTATGCATATACTTCT
	R: GAACATGTCTGCGTATCTC
U6	F: CTCGCTTCGGCAGCACAT
	R: TTTGCGTGTCATCCTTGCG

*RT-qPCR* reverse transcription quantitative polymerase chain reaction, *PCNA* proliferating cell nuclear antigen, *Bax* Bcl2-associated X,* PUMA* BCL2 binding component 3, *PSMD14* proteasome 26S subunit, non-ATPase 14, *TGFBR1* transforming growth factor beta receptor 1, *GAPDH* glyceraldehyde-3-phosphate dehydrogenase, *miR* microRNA

#### Immunofluorescence staining

The rMCs were seeded on slides, permeated, and sealed. The cell slides were incubated with the Smad2/3 antibodies at 4 °C overnight, and then incubated with the FITC-labeled secondary antibody at 25 °C for 60 min. The nuclei were stained by DAPI for 60 s. The staining results were observed under the fluorescence scope and analyzed using the Image J software.

#### Western blot analysis

Total protein from renal tissues and rMCs was collected using the radio-immunoprecipitation assay cell lysis buffer (Beyotime). The protein concentration was determined by the BCA method again. Thereafter, the protein samples were run on SDS–polyacrylamide gel electrophoresis and transferred onto polyvinylidene fluoride membranes (Millipore). The membranes were blocked with 5% bovine serum albumin (Solarbio) for 2 h, and then incubated with the diluted specific primary antibodies at 4 °C overnight, and then with HRP-labeled secondary antibody at 25 °C for 45 min. The protein bands were visualized using an enhanced chemiluminescence (ECL) kit (Millipore), visualized and analyzed using a ChemiDoc MP Imaging System and an Image Lab software, respectively (Bio-Rad, Inc., Hercules, CA, USA).

#### Microarray analysis

The Evs-treated renal tissue and rMCs were used for miRNA microarray analysis. In brief, the fragments were hybridized with the Agilent-Rat microRNA microarray 21.0 (8 * 60 K, design ID: 070,154). The microarray analyses were performed on a miRNA 4.0 platform (Affymetrix, Santa Clara, CA, USA) according to the manufacturer’s instructions. The samples were labeled, hybridized, and washed. Differentially expressed (DE) miRNAs before and after Evs treatment were screened with |Log_2_FoldChange|> 2 as the screening criteria.

#### Dual-luciferase reporter gene assay

The wild-type (wt) 3′-UTR sequence of PSMD14 mRNA containing the putative binding site with miR-378 and the mutant-type (mt) sequence was synthetized by GeneScript Company (Nanjing, Jiangsu, China) and inserted into pGL3 luciferase reporter gene vectors (Promega) to construct pGL3-PSMD14-wt and pGL3-PSMD14-mt vectors. The 293 T cells were seeded in 24-well plates and cultured for 24 h. Well-constructed pGL3 vectors were co-transfected with either miR-378 mimic or mimic control into 293 T cells. After 48 h, the relative luciferase activity was determined using a dual-luciferase assay system (Promega).

#### Co-immunoprecipitation (Co-IP)

Once reaching an 80% confluence, the rMCs were transfected with TGFBR1 or PSMD14 overexpression vectors. After 24 h, the medium was absorbed and 1 mL PBS was added. The cells were collected into 1.5-mL EP tubes, to which 100 μL IP lysis containing phenylmethylsulfonyl fluoride was added, followed by 3 min of ultrasonication in water bath (50%, 3 s/3 s). Next, an appropriate volume of Protein G agarose beads was added, and then the samples were centrifuged at 300 rpm at 4 °C for 1 min to discard the protection fluid. After that, the cells were resuspended in PBS and centrifuged again. Thereafter, 20 μL PBS 2 × SDS protein loading buffer was added, shaken and centrifuged. The immunoprecipitates and input samples were denaturalized in metal bath at 95 °C for 15 min and fully centrifuged. Next, 7 μL supernatant was collected for western blot analysis.

#### Examination of ubiquitination

Overexpression vectors of Flag-TFGBR1, HA-Ub and PSMD14 were transfected into HEK293T cells, respectively. The protein extraction procedure was performed as previously described in the Co-IP assay, and anti-Flag was used for western blot analysis.

#### Statistical analysis

GraphPad 7.0 (GraphPad, La Jolla, CA, USA) and SPSS19.0 (IBM Corp. Armonk, NY, USA) were used for data analysis. Data were collected from three independent experiments and presented as the mean ± standard deviation (SD). Differences were compared by Student’s *t* test (two groups), or by one- or two-way analysis of variance (ANOVA). *P* < 0.05 represents significant difference.

## Results

### UcMSC-Evs alleviate anti-Thy 1.1-induced MsPGN in rats

The western blot analysis confirmed the positive expression of CD73, CD90 and CD105 whereas negative expression of CD34, CD45 and HLA-DR in the acquired ucMSCs. The Oil red O staining and Alizarin red staining results showed that the ucMSCs differentiated into adipoblasts and osteoblasts. After that, the ucMSC-derived particles were collected following gradient centrifugation. The protein concentration was 316.97 μg/mL. According to the Evs identification method issued by MISEV2018 [24], the particles were in round shape or ellipsoid shape under TEM. The Nanoparticle tracking analysis suggested that the diameter of the particles was 68–151 nm with an average diameter of 98.41 nm. In addition, the western blot analysis further confirmed expression of CD63, CD81, ALIX, and TSG101 in the particles, indicating the particles were Evs (data not shown).

**Fig. 1:**
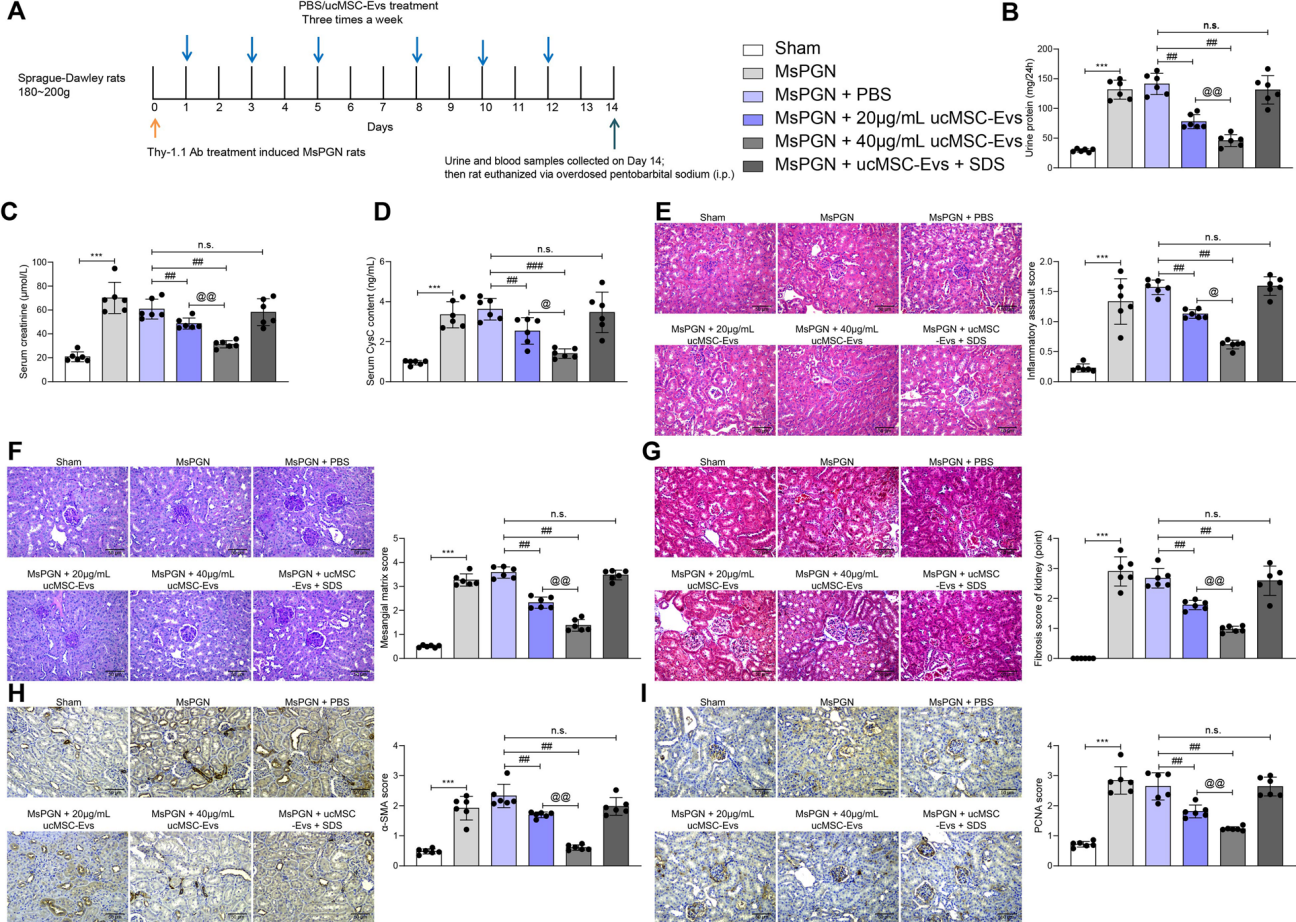
ucMSC-Evs alleviate anti-Thy 1.1-induced MsPGN in rats. **A** a flow chart for MsPGN induction in rats using anti-Thy-1.1; **B**, 24-h urine protein level in rats; **C**, **D**, serum creatinine (**C**) and Cys-C (**D**) levels in rats; E, pathological changes in mouse renal tissues observed by HE staining; **F**, the concentration of glycogen in the renal tissues in MsPGN rats determined by PAS staining; **G**, fibrosis in rat renal tissues determined by Masson's trichrome staining; **H**, **I** staining intensity of α-SMA (**H**) and PCNA (**I**) in renal tissues determined by IHC staining. N = 6 in each group. Data were expressed as the mean ± SD. Each spot indicates a single rat. Data were analyzed by one-way ANOVA followed by Tukey’s multiple comparison test. ***p < 0.001 versus sham group; ##*p* < 0.01, ###*p* < 0.001 versus MsPGN + PBS; @ *p* < 0.05, @@ *p* < 0.01 versus MsPGN + 20 μg/mL ucMSC-Evs; n.s. represents no significance

The roles of Evs in MsPGN has not been concerned before. The extracted Evs were diluted and administrated into model rats at 0 μg/mL/kg (PBS), 20 μg/mL/kg or 40 μg/mL/kg, and another group of rats were treated with SDS-treated 40 μg/mL/kg Evs (Fig. 1A). It was found that after anti-Thy-1.1 induction, the urine protein level in 24 h, and the concentration of serum creatinine and Cys-C were increased. Both levels were blocked following Evs treatment in a dose-dependent manner, since 40 μg/kg showed a better inhibiting function. However, this inhibition was blocked by when the Evs were treated with SDS (Fig. 1B–D). After that, the rats were euthanized, and the right renal tissues were collected for histological staining. The HE staining observed aberrant hyperplasia of MCs and significant immune cell infiltration in renal tissues after anti-Thy-1.1 administration (Fig. 1E). The PAS staining suggested that the concentration of glycogen in the renal tissues in MsPGN rats was increased (Fig. 1F), accompanying with increased fibrosis in kidney tissues according to the Masson's trichrome staining (Fig. 1G). In addition, the IHC staining intensity of PCNA and α-SMA in renal tissues were increased (Fig. 1H, I). Further, these pathological changes in renal tissues were significantly alleviated by usMSC-Evs in a dose-dependent manner. But the effects of Evs were almost completely suppressed by SDS (Fig. 1E–I).

### UcMSC-Evs suppress PDGF-induced proliferation of rMCs

**Fig. 2:**
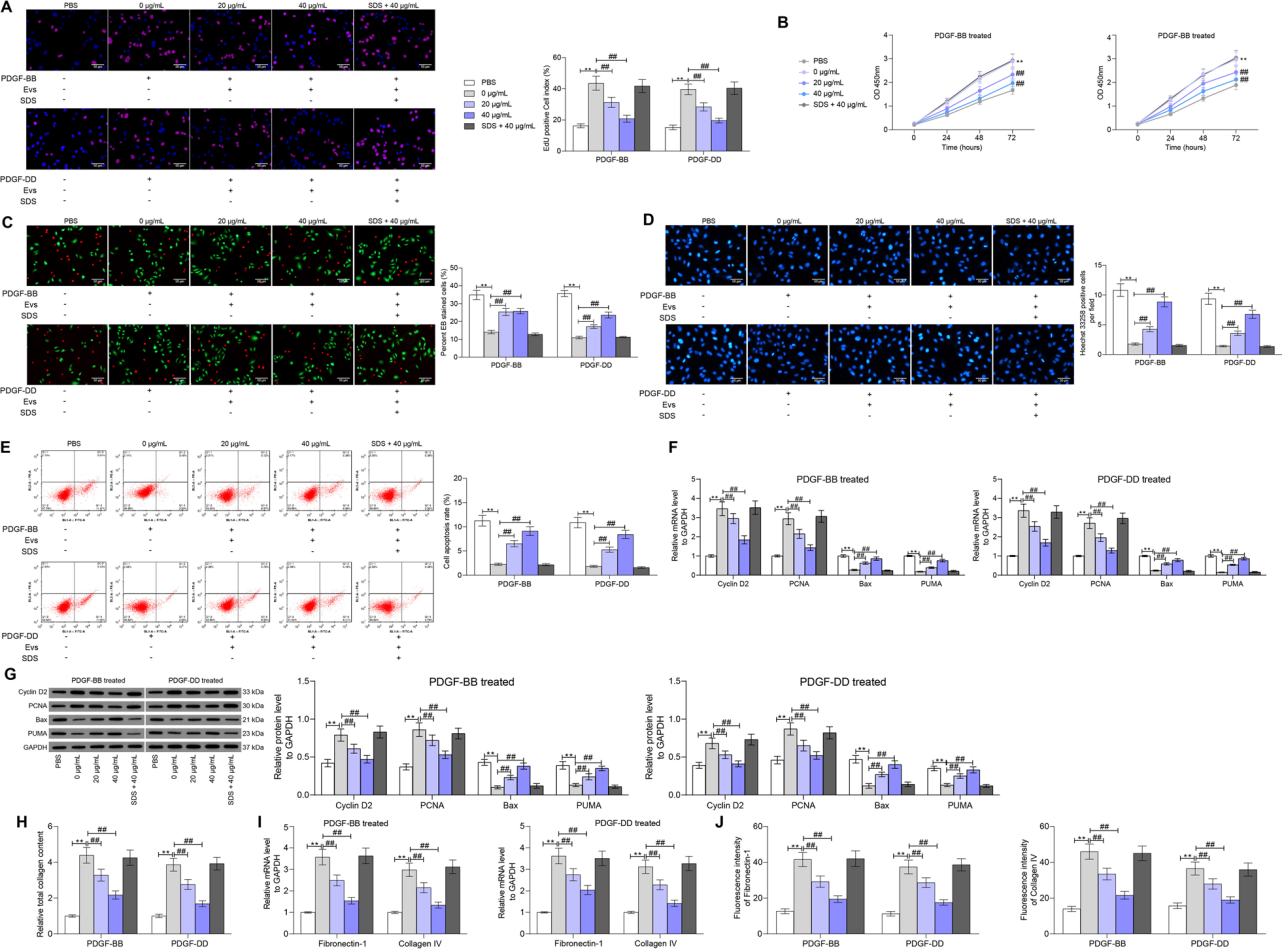
ucMSC-Evs suppress PDGF-induced proliferation of rMCs. **A** proliferation of rMCs determined by EdU labeling; **B** viability of cells determined using a CTG kit; **C** portion of dead cells confirmed by EB fluorescence staining; **D** apoptosis of cells determined by Hoechst 33,258 staining; **E**, apoptosis rate of cells determined by flow cytometry; **F**, **G** mRNA (**F**) and protein (**G**) expression of Cyclin D2, PCNA, Bax and PUMA in cells determined by RT-qPCR and western blot analysis, respectively; **H**, total collagen concentration in rMCs determined by collagen kits; **I**,** J**, expression of fibronectin-1 and collagen IV in cells determined by RT-qPCR and immunofluorescence staining. Data were expressed as mean ± SD from at least three independent experiments. Data were analyzed by two-way ANOVA followed by Tukey’s multiple comparison test. **p < 0.01, ***p < 0.001 versus PBS group; ##*p* < 0.01, ###*p* < 0.001 versus 0 μg/mL group

To further investigate the functions of ucMSC-Evs in MsPGN, the PDGF-BB/DD-treated rMCs were further treated with ucMSC-Evs treatment at different doses (0, 20, or 40 μg/mL), or further treated with SDS-treated ucMSC-Evs. rMCs treated with PBS (without PDGF-BB/DD treatment) were set as the blank group. The EdU labeling results suggested that the number of EdU-positive cells was significantly increased after PDGF treatment (Fig. 2A). The cell viability, according to the CTG assay, was enhanced as well (Fig. 2B). In addition, the apoptosis rate of rMCs, according to the AO/EB staining, Hoechst 33,258 staining and flow cytometry, was significantly reduced by PDGF (Fig. 2C–E). From the cytokine perspective, we found that PDGF treatment increased the expression of proliferation-related factors (Cyclin D2 and PCNA) whereas decreased the expression of apoptosis-related factors (Bax and PUMA) (Fig. 2F, G). The changes induced by PDGF were blocked by ucMSC-Evs in a dose-dependent manner. However, the anti-proliferation function of Evs was diminished by SDS again (Fig. 2A–G).

Since aberrant proliferation of MCs can lead to an increase in the concentration of ECM and the further MsPGN progression [25], we therefore examined total collagen concentration in rMCs. It was observed that the collagen concentration was increased by PDGF but then suppressed by ucMSC-Evs (Fig. 2H). In addition, the mRNA and protein expression of fibronectin-1 and collagen IV in cells was increased by PDGF and then inhibited by Evs as well (Fig. 2I, J). Similarly, the blockage of Evs on PDGF-induced collagen deposition was further diminished by SDS (Fig. 2H–J).

### UcMSC-Evs suppress activation of the TGF-β1/Smad2/3 signaling pathway in anti-Thy-1.1-indued rats and PDGF-treated cells

**Fig. 3:**
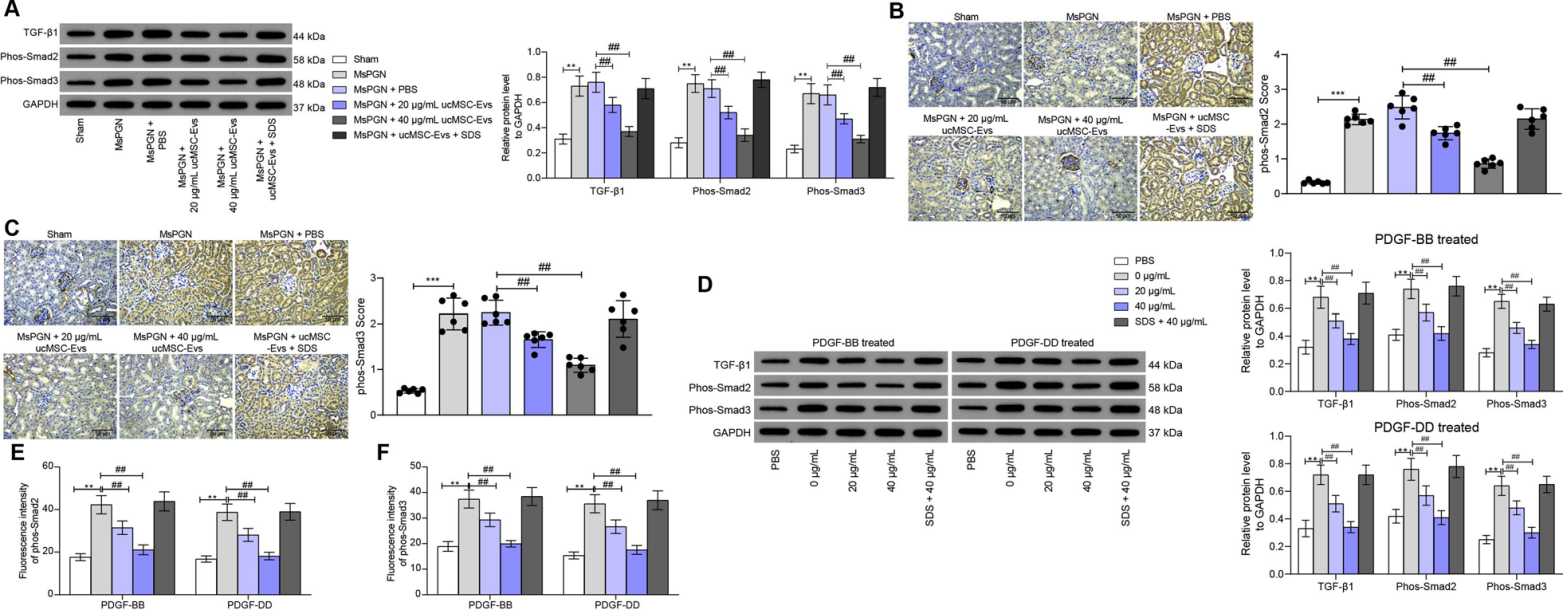
ucMSC-Evs suppress activation of the TGF-β1/Smad2/3 signaling pathway in anti-Thy-induced rats and PDGF-treated cells. **A** expression of TGF-β1 and phosphorylation of Smad2/3 in rat renal tissues examined by western blot analysis; **B**, **C** phos-Smad2/3-positive scores in rat renal tissues detected by IHC staining; **D** expression of TGF-β1 and phosphorylation of Smad2/3 in rMCs examined by western blot analysis; **E**, **F** subcellular localization of Smad2/3 in rMCs determined by immunofluorescence staining. In animal models, n = 6 in each group. Each spot indicates a single rat. Data were expressed as mean ± SD from at least three independent experiments. Data were analyzed by one- two-way ANOVA, followed by Tukey’s multiple comparison test. **p < 0.01 versus sham group or PBS group; ##*p* < 0.01 versus MsPGN + PBS or 0 μg/mL group

The TGF-β1/Smad2/3 is frequently linked to MC proliferation and ECM production [12, 26]. Here, we tested if this signaling is implicated in the anti-MsPGN events by ucMSC-Evs. According to the western blot analysis, the expression of TGF-β1 and the phosphorylation of Smad2/3 were increased in the rat renal tissues after anti-Thy-1.1 induction. Importantly, these changes were reversed by ucMSC-Evs (Fig. 3A). In line, the number of phos-Smad2/3-positive cells was increased according to the IHC staining (Fig. 3B, C). The expression of TGF-β1 and the phosphorylation of Smad2/3 were increased in rMCs after PDGF-BB/DD treatment (Fig. 3D). In addition, the immunofluorescence staining suggested that the nuclear translocation of Smad2/3 in rMCs was increased after PDGF-BB/DD treatment (Fig. 3E, F).

### UcMSC-Evs carry miR-378 to negatively regulate PSMD14

**Fig. 4:**
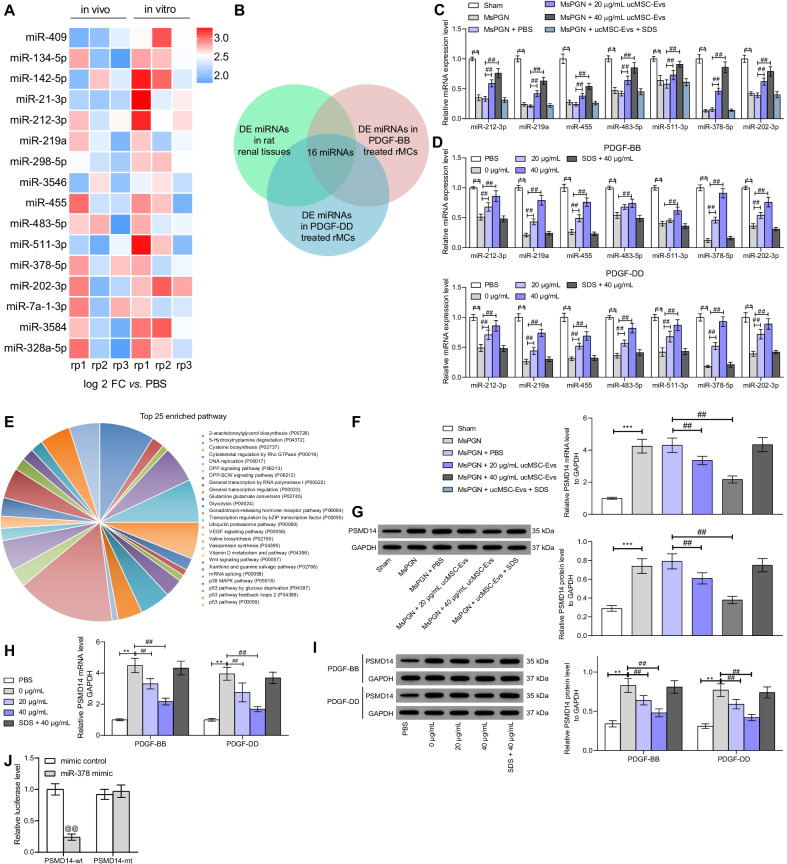
ucMSC-Evs carry miR-378 to negatively regulate PSMD14. **A** a heatmap for the top 30 DE miRNAs in rat renal tissues and rMCs before and after 40 μg/mL Evs treatment according to a miRNA microarray analysis; **B** selection of the shared DE miRNAs in tissues and cells; C-D, expression of 7 upregulated miRNAs in renal tissues (**C**) and rMCs (**D**) after ucMSC-Evs treatment determined by RT-qPCR; **E** a functional classification analysis among the target mRNAs of miR-378 performed on the Panther System; **F**–**G** mRNA (**F**) and protein (**G**) expression of PSMD14 in rat renal tissues determined by RT-qPCR and western blot analysis, respectively; **H**, **I** mRNA (**H**) and protein (**I**) expression of PSMD14 in rMCs determined by RT-qPCR and western blot analysis, respectively; **J** binding relationship between miR-378 and PSMD14 determined using the luciferase reporter gene assay. In animal models, n = 6 in each group. Data were expressed as mean ± SD from at least three independent experiments. Data were analyzed by one- or two-way ANOVA followed by Tukey’s multiple comparison test. **p < 0.01 versus sham group or PBS group; ##*p* < 0.01versus PDGF-BB group or 0 μg/mL group; @@ *p* < 0.01 versus mimic control

miRNAs are the most common cargoes delivered by Evs. Therefore, miRNA microarray analyses were performed to identify the DE miRNAs both in renal tissues and in rMCs after 40 μg/mL Evs treatment. The detailed information of DE miRNAs is presented in Additional file 1: Tables. A Venn diagram plotted 16 shared DE miRNAs in these three microarray analysis sets (Fig. 4A, B). Among them, seven miRNAs (miR-212-3p, miR-219a, miR-455, miR-483-5p, miR-511-3p, miR-378-5p, and miR-202-3p) were suggested to be upregulated in tissues or cells after 40 μg/mL treatment. Importantly, RT-qPCR suggested that miR-378 was the miRNA with the highest fold of change after Evs treatment both in tissues and rMCs (Fig. 4C, D). We therefore surmised that miR-378 is involved in events mediated ucMSC-Evs. To further explore the downstream mechanism, we predicted the target mRNAs of miR-378 on RNA22 (https://cm.jefferson.edu/rna22/). A total of 3985 candidate target genes of miR-378 were predicted. Later, a functional classification analysis based on these candidate genes was performed in the Panther Website (http://www.pantherdb.org/), which suggested that the miR-378-targeted genes, including PSMD14, were mainly enriched in the ubiquitin proteasome pathway (Fig. 4E). The reason why we focused on PSMD14 was that it was reported to trigger the deubiquitination to maintain the stability of TGF-β receptors (TGFBR1 and TGFBR2) [14]. Therefore, there might be a PSMD14-TGF-β interaction in MsPGN. Thereafter, we investigated the expression of PSMD14 in rat renal tissues and rMCs. The mRNA and protein expression of PSMD14 in tissues and cells was significantly increased after anti-Thy-1.1 or PDGF-BB/DD treatment. Importantly, the PSMD14 expression was suppressed by Evs (Fig. 4F–I). To validate the binding between miR-378 and PSMD14, a luciferase reporter gene assay was performed, which suggested that miR-378 mimic significantly reduced the luciferase activity of PSMD14-wt in 293 T cells. However, the luciferase activity in cells transfected with mimic control or PSMD14-mt was not significantly changed (Fig. 4J).

### Reduction of miR-378 blocks the functions of ucMSC-Evs

**Fig. 5:**
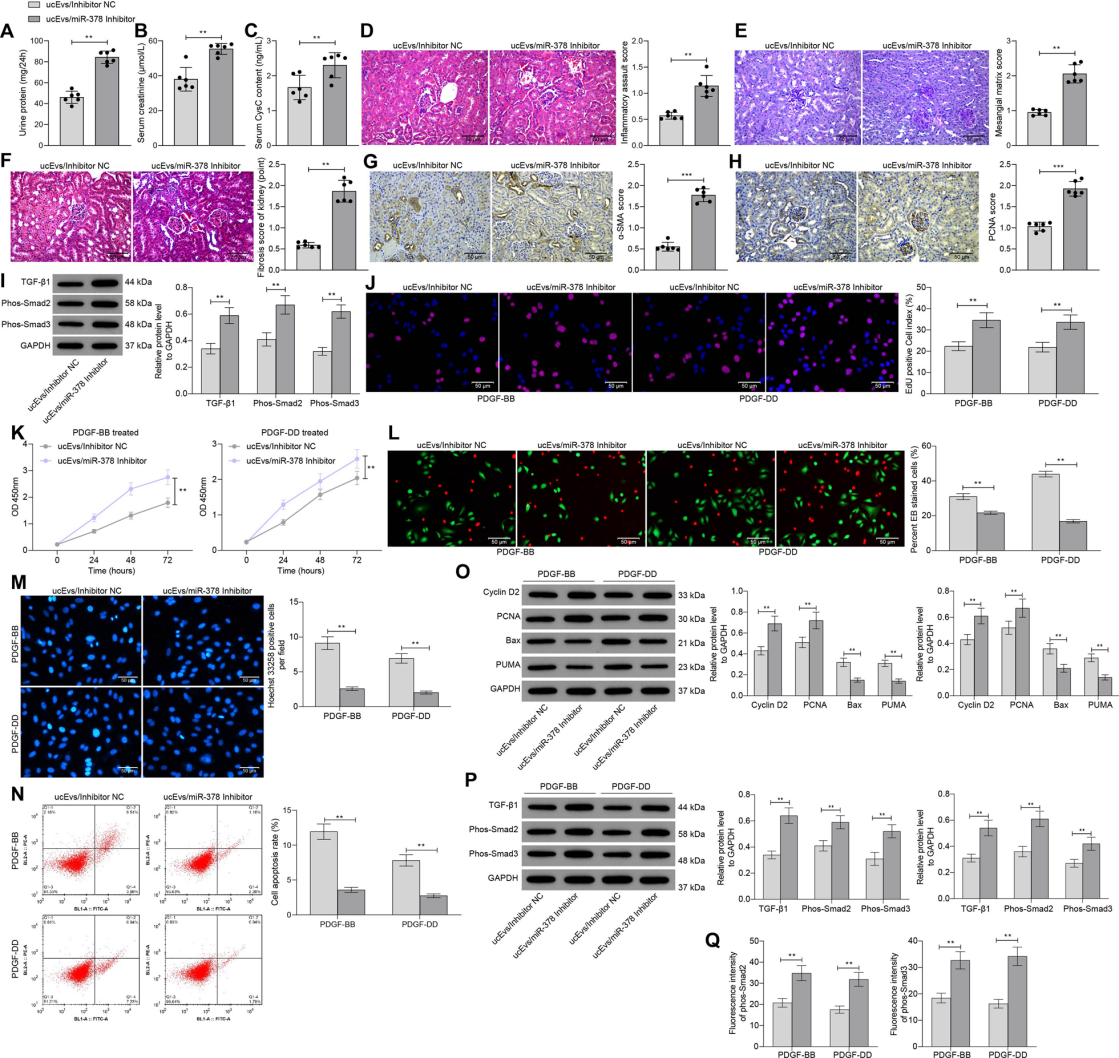
Reduction of miR-378 blocks the functions of ucMSC-Evs. **A** 24-h urine protein level in rats; **B**,** C**, serum creatinine (**B**) and Cys-C (**C**) levels in rats; **D** pathological changes in mouse renal tissues observed by HE staining; **E**, the concentration of glycogen in the renal tissues in MsPGN rats determined by PAS staining; **F**, fibrosis in rat renal tissues determined by Masson's trichrome staining; **G**, **H** staining intensity of α-SMA (**G**) and PCNA (**H**) and in renal tissues determined by IHC staining, **I**, expression of TGF-β1 and phosphorylation of Smad2/3 in rat renal tissues determined by western blot analysis; **J** proliferation of rMCs determined by EdU labeling; **K** viability of cells determined using a CTG kit; **L** portion of dead cells confirmed by EB fluorescence staining; **M** apoptosis of cells determined by Hoechst 33,258 staining; **N**, apoptosis rate of cells determined by flow cytometry; **O** protein expression of Cyclin D2, PCNA, Bax and PUMA in cells determined by western blot analysis; **P** expression of TGF-β1 and phosphorylation of Smad2/3 in rMCs determined by western blot analysis; **Q** expression of phos-Smad2/3 in cells determined by immunofluorescence staining. In animal models, n = 6 in each group. Each spot indicates a single rat. Data were expressed as mean ± SD from at least three independent experiments. Data were analyzed by the unpaired *t* test or two-way ANOVA followed by Tukey’s multiple comparison test. **p < 0.01, ***p < 0.001 versus Evs/Inhibitor control group

To validate the involvement of miR-378 in the protective events mediated by ucMSC-Evs, miR-378 inhibitor or inhibitor NC was administrated into ucMSCs, and the corresponding Evs, named ucEvs/miR-378 inhibitor or ucEvs/inhibitor NC, were collected. The protein concentration of the extracted Evs was 335.81 μg/mL. The Thy-1.1-treated rats and the PDGF-BB/DD-treated cells were further administrated with ucEvs/miR-378 inhibitor or ucEvs/inhibitor NC. Down-regulation of miR-378 in Evs increased the 24-h urine protein level and the concentrations of serum creatinine and Cys-C in rats (Fig. 5A–C). In addition, the glycogen concentration and fibrosis in rat renal tissues was increased, along with increased IHC staining intensity of PCNA and α-SMA (Fig. 5D–H). Moreover, inhibition of miR-378 promoted the activation of TGF-β1/Smad2/3 signaling pathway (Fig. 5I).

In PDGF-treated rMCs, the number of EdU-positive cells, and the viability of cells were increased and the portion of dead cells was decreased after miR-378 inhibition in the Evs (Fig. 5J, K). Moreover, the apoptosis of rMCs was reduced (Fig. 5L–N). Downregulation of miR-378 in the ucMSC-Evs led to an increase in the expression of Cyclin D2 and PCNA while a decline in the expression of Bax and PUMA (Fig. 5O). In addition, the activity of TGF-β1/Smad2/3 signaling pathway was increased in cells after miR-378 inhibition as well, and the nuclear translocation of Smad2/3 was promoted accordingly (Fig. 5P, Q).

### PSMD14 promotes TGFBR1 stability through deubiquitination modification

**Fig. 6:**
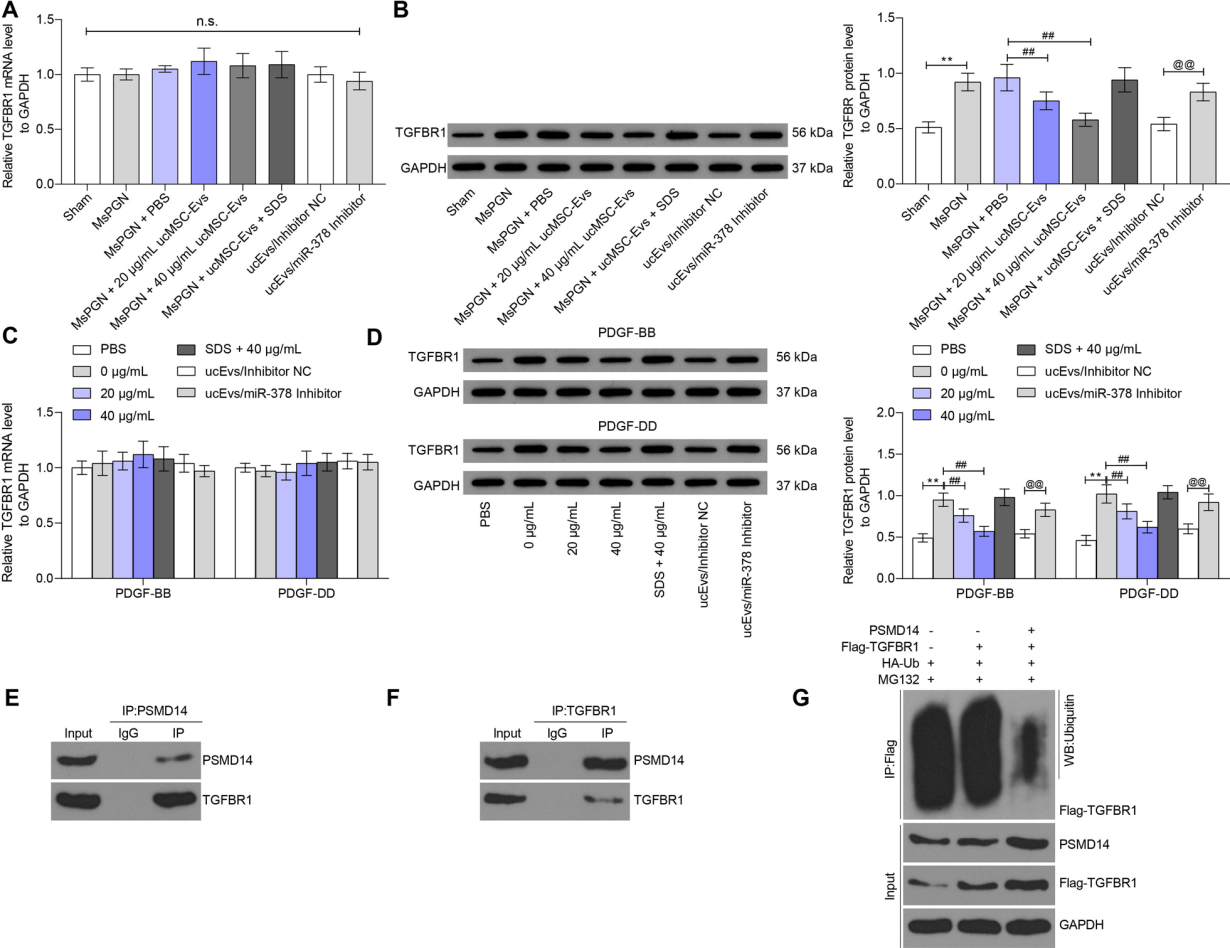
PSMD14 promotes TGFBR1 stability through deubiquitination modification. **A**, **B** mRNA (**A**) and protein (**B**) expression in rat renal tissues determined by RT-qPCR and western blot analysis, respectively; **C**, **D** mRNA (**C**) and protein (**D**) expression in rMCs determined by RT-qPCR and western blot analysis, respectively; **E**, **F** binding relationship between PSMD14 and TGFBR1 validated through a Co-IP assay; **G** ubiquitination level of TGFBR1 in 293 T cells after overexpressing Flag-TGFBR1, HA-Ub and PSMD14 in cells. Data were expressed as mean ± SD from at least three independent experiments. Data were analyzed by one- or two-way ANOVA followed by Tukey’s multiple comparison test. **p < 0.01, ***p < 0.001 versus or PBS group; ##*p* < 0.01 versus MsPGN + PBS group or 0 μg/ml group; @@ *p* < 0.01 versus Evs/Inhibitor NC group; n.s. represents no significance

As mentioned before, PSMD14 might activate the TGF-β signaling pathway through the deubiquitination modification of TGFBRs, and we observed that the TGFβ1/Smad2/3 signaling pathway was activated in rat renal tissues and rMCs after anti-Thy-1.1 or PDGF administration. Here, we further explored the expression of TGFBR1 in tissue and cell models. Importantly, it was found that the mRNA expression of TGFBR1 showed no significant changes, while the protein expression of TFGBR1 in renal tissues and rMSCs was significantly declined by ucMSC-Evs in a dose-dependent manner and then recovered upon miR-378 inhibition (Fig. 6A–D).

To further validate if PSMD14 regulates the stability of TGFBR1, we first confirmed the binding relationship between PSMD14 and TGFBR1 in rMSCs through a Co-IP assay. An enrichment of TFGBR1 fragments was found in the immunoprecipitates combined by anti-PSMD14, and accordingly an enrichment of PSMD14 fragments was found in the immunoprecipitates combined by anti-TGFBR1 (Fig. 6E, F). In addition, overexpression of Flag-TGFBR1, HA-Ub and PSMD14 was introduced in 293 T cells, by which we found that overexpression of PSMD14 suppressed the ubiquitination of TGFBR1 in cells (Fig. 6G).

### Silencing of PSMD14 blocks PDGF-induced abnormal proliferation of rMCs

**Fig. 7:**
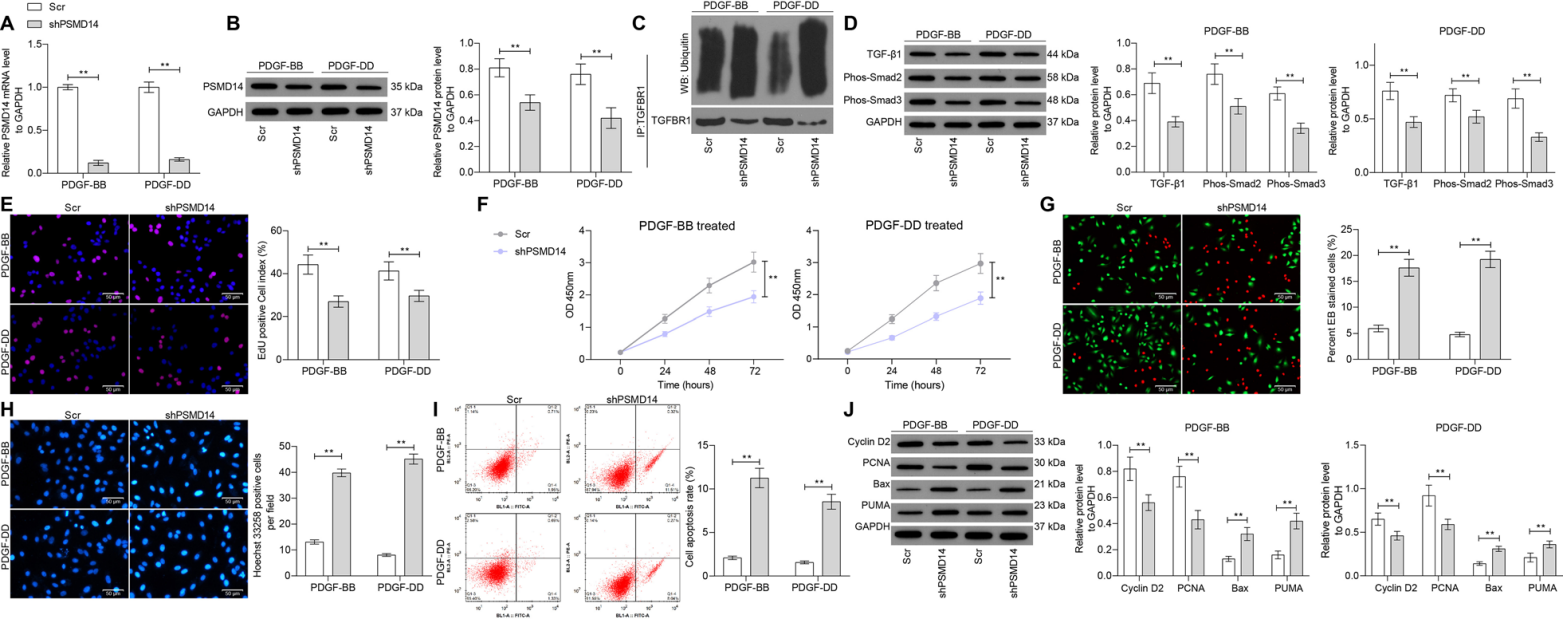
Silencing of PSMD14 blocks PDGF-induced abnormal proliferation of rMCs. **A**, **B** mRNA (**A**) and protein expression of PSMD14 in rMCs after shPSMD14 transfection determined by RT-qPCR and western blot analysis, respectively; **C** determination of the ubiquitination level of TGFBR1; **D** expression of TGF-β1 and phosphorylation of Smad2/3 in rMCs determined by western blot analysis; **E** proliferation of rMCs determined by EdU labeling; **F**, viability of cells determined using a CTG kit; **G** portion of dead cells detected by EB fluorescence staining; **H** apoptosis of cells determined by Hoechst 33,258 staining; **I**, apoptosis rate of cells determined by flow cytometry; **J** protein expression of Cyclin D2, PCNA, Bax and PUMA in cells determined by western blot analysis. Data were expressed as the mean ± SD from at least three independent experiments. Data were analyzed by one-way ANOVA followed by Tukey’s multiple comparison test. **p < 0.01 versus Scr group

To further explore the role of PSMD14 in MsPGN progression, two short hairpin (sh) RNAs targeting PSMD14 (shPSMD14) were introduced in rMCs, and the successful transfection was detected by RT-qPCR and western blot analysis (Fig. 7A, B). Next, the expression of TGFBR1 and the activation of the TGFBR1-TGF-β1/Smad2/3 signaling pathway in rMCs was determined. It was found that the ubiquitination of PDGF was significantly increased upon PSMD14 silencing (Fig. 7C), and the following TGF-β1 expression as well as Smad2/3 phosphorylation were suppressed (Fig. 7D). In terms of cell behaviors, it was found that the proliferative activity of cells was suppressed (Fig. 7E, F), whereas the apoptosis of cells was increased (Fig. 7G–I) after PSMD14 silencing. Correspondingly, the expression of proliferation-related factors PCNA and Cyclin D2 in cells was significantly decreased, whereas the expression of apoptosis-related Bax and PUMA was increased after PSMD14 knockdown (Fig. 7J).

### Overexpression of PSMD14 blocks the anti-protective role of ucMSC-Evs in rMCs

**Fig. 8:**
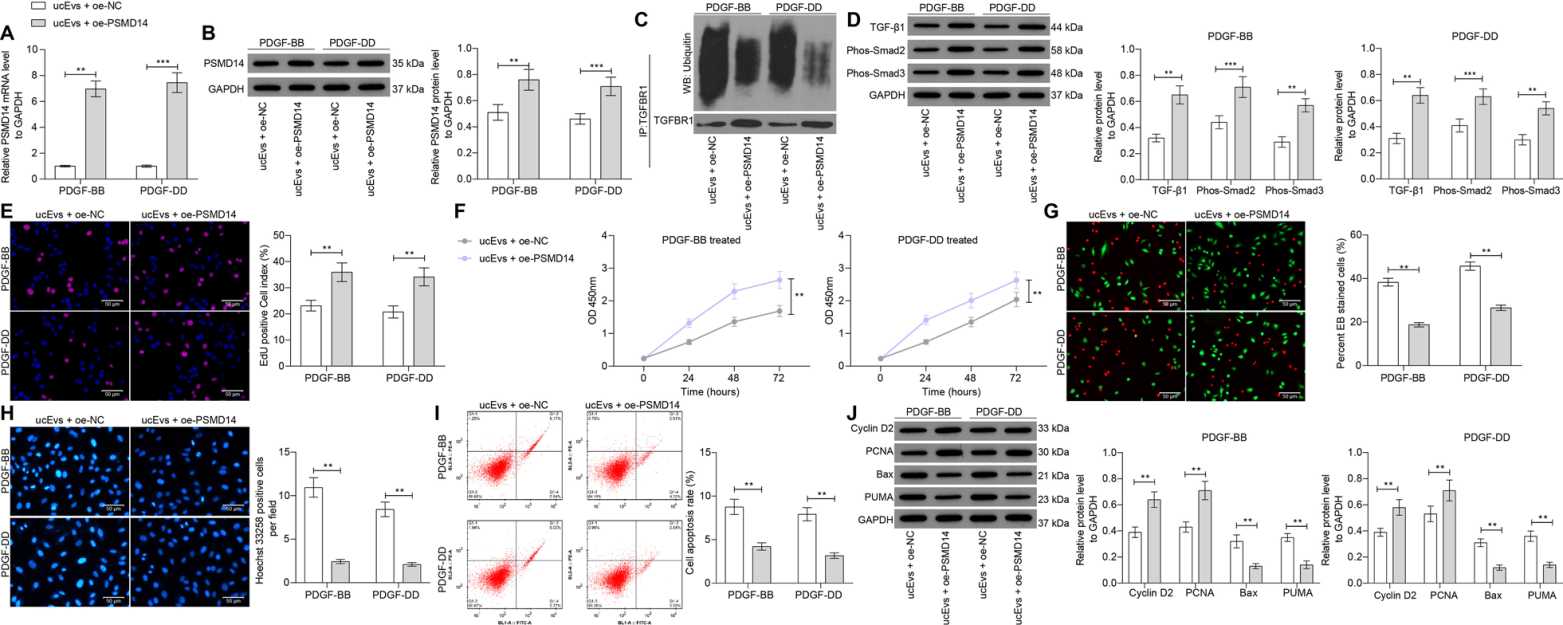
Overexpression of PSMD14 blocks the protective roles of ucMSC-Evs against PDGF-induced proliferation of rMCs. **A**, **B** mRNA and protein expression PSMD14 in cells after oe-PSMD14 transfection determined by RT-qPCR (**A**) and western blot analysis (**B**), respectively; **C** determination of the ubiquitination level of TGFBR1; **D** expression of TGF-β1 and phosphorylation of Smad2/3 in rMCs determined by western blot analysis; **E** proliferation of rMCs determined by EdU labeling; **F** viability of cells determined using a CTG kit; **G** ratio of live to dead cells confirmed by AO/EB double fluorescence staining; **H** apoptosis of cells determined by Hoechst 33258 staining; **I** apoptosis rate of cells determined by flow cytometry; **J** levels of the apoptosis-related proteins determined by western blot analysis. Data were expressed as mean ± SD from at least three independent experiments. Data were analyzed by two-way ANOVA followed by Tukey’s multiple comparison test. **p < 0.01, ***p < 0.001 versus ucEvs + oe-NC group

Following the findings above, overexpression of PSMD14 was introduced in cells after ucMSC-Evs treatment, and the successful transfection was determined by RT-qPCR and western blot analysis (Fig. 8A, B). This significantly inhibited the ubiquitination and degradation of TGFBR1 (Fig. 8C). Therefore, the expression of TGF-β1 as well as the phosphorylation of Smad2/3 in cells were increased (Fig. 8D). In this condition, the proliferation of cells was significantly increased (Fig. 8E, F), whereas the apoptosis of cells was decreased (Fig. 8G–J).

## Discussion

Comprehensive measures such as corticosteroids, antiplatelet agents, immunosuppressive agents, and antilipemic agents have been used to alleviate glomerulosclerosis; however, there is no effective regimen to date for MsPGN treatment [27]. Due to their potent roles in inflammation suppression and tissue regeneration, MSCs have aroused as a subject of research in kidney diseases [28–30]. Here, we report that ucMSC-Evs-delivered miR-378 suppresses PSMD14-mediated TGFBR1 stability and inactivates the TGF-β1/Smad2/3 signaling pathway, therefore suppressing renal tissue hyperplasia and fibrosis as well as rMC proliferation to ameliorate MsPGN.

The protective potential of MSCs and their Evs have been summarized in several kidney impairments such as ischemic acute kidney injury, lupus and diabetic nephropathies, and renal transplantation [28, 31]. After identification of ucMSCs and the extracted Evs by their specific biomarkers (CD73, CD90 and CD105 for MSCs) (CD63, CD81 and ALIX for Evs), the ucMSC-Evs were administrated into the anti-Thy-1.1-treated rats and PDGF-treated rMCs. Consequently, the MsPGN symptoms in rat renal tissues, and the aberrant proliferation of rMCs were suppressed by ucMSC-Evs in a dose-dependent manner. MSCs have presented repair effects on podocyte damage in mice with IgA nephropathy, including relieving fibrosis degree and alleviating hematuresis and proteinuria [32]. Through the delivery of Evs, MSCs also showed significant therapeutic efficiency for cisplatin-induced acute kidney injury [33]. A recent study by Wang et al*.* suggested that ucMSC-derived exosomes alleviated proteinuria, glomerulus injury, and fibrosis in mice with diabetic nephropathy and reduced deposition of fibronectin and collagen I in MCs [26], which were partially in agreement with our experimental results. The potential of MSC-Evs in clinical use has been also demonstrated in a previous report, in which administration of MSC-Evs improved symptom, reduced serum creatinine, and improved urinary albumin creatinine ratio without significant side-effects in patients with long-term chronic kidney disease [34].

The study by Wang et al*.* also suggested that reduction of the TGF-β1/Smad2/3 signaling pathway is responsible for the MSC-Evs-reduced renal fibrosis in mouse models with diabetic nephropathy [26]. As we mentioned before, this signaling is a major profibrotic factor that can promote the production of ECM and the consequent renal fibrosis [12]. Similarly, the TGF-β1/Smad2/3 signaling has been reported to be correlated with renal fibrosis and aberrant hyperplasia in diabetic nephropathy [26]. We then wondered if this is also applied in the MsPGN models. According to the western blot analysis, it was found that the expression of TGF-β1 and phosphorylation of Smad2/3 were significantly increased in model rats and rMSCs but then suppressed by the ucMSC-Evs, indicating that the Evs may suppress the TGF-β1/Smad2/3 signaling pathway to ameliorate MsPGN and block rMC proliferation. But the regulatory work remained unclear.

Evs are well known to exert their functions through carrying different cargoes [35, 36]. We found that the protective effects of ucMSC-Evs on rats and rMCs were blocked by SDS, indicating that the maintenance of the membrane structure for delivery of the specific ‘cargoes’ is essential for ucMSC-Evs. Studies have reported that MSC-derived Evs contain specific miRNAs such as miR-34c-5p [37] and miR-29 [38]. In the present study, the miRNA microarray analyses identified miR-378 as the miRNA with the highest fold of upregulation in tissues and cells after Evs treatment. The inhibiting role of miR-378 in mesangial hypertrophy, ECM production and renal fibrosis has been documented once [13]. Likewise, miR-378 was responsible for the protective role of atragaloside against diabetic nephropathy [39]. Moreover, miR-378-containing Evs were linked to several bio-functional processes such as wound healing [40] or cancer progression [41]. In this work, the involvement of miR-378 in the protective events mediated by ucMSC-Evs was confirmed by the rescue experiments, in which downregulation of miR-378 blocked the anti-MsPGN functions of the Evs either in vivo and in vitro.

When exploring the downstream targets of miR-378, PSMD14 (also known as RPN11 or POH1), a deubiquitinating enzyme has a fundamental function to suppress protein degradation and regulate multiple biological processes [42–44], attracted our attention. Especially when PSMD14 has been reported to maintain the stability of TGFBRs through deubiquitination modification [14]. We therefore wondered whether PSMD14 can stabilize TGFB1 and regulate the TGF-β1/Smad2/3 activity to modulate MsPGN. Thereafter, a direct binding relationship between PSMD14 and TGFB1 was identified. The protein level of TGFBR1, rather than the mRNA expression, was significantly decreased tissues cells by ucMSC-Evs but recovered after miR-378 downregulation. Artificial silencing of PSMD14 by shRNAs inactivated the TGF-β1/Smad2/3 pathway and blocked PDGF-induced proliferation of rMCs, which showed similar effects of UcMSC-Evs. This body of evidence suggested that suppression of PSMD14 and TGFBR1 and the inactivation of the TGF-β1/Smad2/3 signaling pathway are, at least partly, involved in the anti-MsPGN and anti-proliferative effects mediated by UcMSC-Evs-carried miR-378.

## Conclusion

**Fig. 9:**
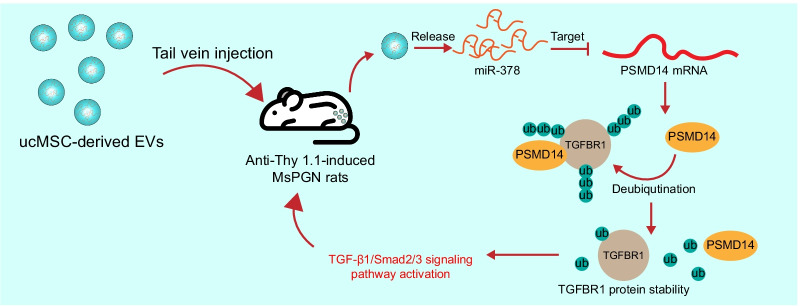
A graphic abstract. Tail vein injection of ucMSC-Evs protect rats against anti-Thy-1.1-induced MsPGN through the release of miR-378. miR-378 suppresses the expression of PSMD14 to restore the ubiquitination and degradation of TGFBR1 and inactivate the TGF-β1/Smad2/3 signaling pathway

In summary, this study validated the protective roles of ucMSC-Evs against anti-Thy-1.1-induced MsPGN in rats and PDGF-induced rMC proliferation through the delivery of miR-378 and the subsequent inhibition of the PSMD14/TGF-β1/Smad2/3 axis (Fig. 9). However, there may more candidate genes and downstream pathways mediated by miR-378 that are involved in the progression of MsPGN. We would like to explore more possible molecular mechanisms in our future studies. Nevertheless, the findings of the present study may offer novel insights into the control of MsPGN and other chronic kidney impairments.

## Supplementary Information

**Additional file 1: Supplementary Tables.** DE miRNAs in rat renal tissues and rMCs after ucMSC-Evs treatment.

## Data Availability

All data in our study are available upon request.

## Abbreviations

ANOVA: Analysis of variance
AO/EB: Acridine orange/ethidium bromide
BCA: Bicinchoninic acid
Co-IP: Co-immunoprecipitation
DMEM: Dulbecco's modified Eagle's medium
ECM: Extracellular matrix
ESRD: End-stage renal disease
Evs: Extracellular vesicles
FITC: Fluorescein isothiocyanate
IgG: Immunoglobulin G
MCs: Mesangial cells
MSCs: Mesenchymal stem cells
MsPGN: Mesangial proliferative glomerulonephritis
PCNA: Proliferating cell nuclear antigen
PDGF: Platelet-derived growth factors
PSMD14: Proteasome 26S subunit, non-ATPase 14
rMCs: Rat mesangial cells
TEM: Transmission electron microscope
TGF-β1: Transforming growth factor-β1
uc: Umbilical cord
ucMSCs: Umbilical cord mesenchymal stem cells
ucMSC-Evs: UcMSCs-derived Evs
α-SMA: α-Smooth muscle actin
