# Supplementary material for: Ameliorating role of microRNA-378 carried by umbilical cord mesenchymal stem cells-released extracellular vesicles in mesangial proliferative glomerulonephritis
Source: Cell Commun Signal. 2022 Mar 9;20:28. doi: 10.1186/s12964-022-00835-1 (PMC8905735; doi:10.1186/s12964-022-00835-1)
Supplement: Supplementary file 2 — Additional file 1: Supplementary Tables. DE miRNAs in rat renal tissues and rMCs after ucMSC-Evs treatment. [file 12964_2022_835_MOESM2_ESM.docx]

DE miRNAs in EVs-treated MsPGN rats

| Symbol | FC | Adj p value | Symbol | FC | Adj p value |
| --- | --- | --- | --- | --- | --- |
| miR-409 | 2.29548 | 5.45E-10 | miR-34c-5p | -6.11673 | 7.15E-08 |
| miR-134-5p | 2.15762 | 1.70E-09 | miR-328b-3p | -2.64273 | 7.16E-08 |
| miR-142-5p | 2.25717 | 1.74E-09 | miR-30c-2-3p | -2.22863 | 7.52E-08 |
| miR-21-3p | 2.48456 | 2.45E-09 | miR-210-3p | -2.04993 | 7.78E-08 |
| miR-212-3p | 3.7639 | 3.00E-09 | miR-99a-5p | -3.05168 | 7.85E-08 |
| miR-219a | 2.94583 | 3.15E-09 | miR-203a-3p | -2.25978 | 8.42E-08 |
| miR-298-5p | 1.45052 | 4.50E-09 | miR-30c-1-3p | -2.29865 | 9.20E-08 |
| miR-3546 | 3.30921 | 4.56E-09 | miR-139-3p | -3.62135 | 1.01E-07 |
| miR-455 | 2.84619 | 6.95E-09 | miR-329-3p | -2.69729 | 1.01E-07 |
| miR-483-5p | 1.93748 | 9.39E-09 | miR-1-5p | -1.83303 | 1.11E-07 |
| miR-511-3p | 2.5106 | 1.04E-08 | miR-23b-3p | -2.66553 | 1.13E-07 |
| miR-378-5p | 3.315668 | 1.08E-08 | miR-3584-3p | -3.10555 | 1.28E-07 |
| miR-202-3p | 2.59141 | 1.12E-08 | miR-30e-3p | 2.434157 | 1.32E-07 |
| miR-7a-1-3p | 1.30735 | 1.27E-08 | miR-652-3p | 3.569384 | 1.34E-07 |
| miR-3584 | 2.6813 | 1.51E-08 | miR-374-5p | -3.18389 | 1.37E-07 |
| miR-328a-5p | 1.92475 | 1.51E-08 | miR-15a-5p | -2.31562 | 1.49E-07 |
| miR-193a-3p | -2.86324 | 1.72E-08 | miR-3552 | -1.90062 | 1.50E-07 |
| miR-672-5p | -2.53936 | 2.26E-08 | miR-142-3p | -2.76923 | 1.63E-07 |
| miR-29b-3p | -2.6954 | 4.26E-08 | miR-352 | -1.81289 | 1.79E-07 |
| miR-342-5p | -3.50438 | 4.28E-08 | miR-30d-5p | 2.755144 | 2.06E-07 |
| let-7d-3p | -2.56965 | 4.78E-08 | miR-300-3p | -2.14717 | 2.09E-07 |
| miR-15b-5p | -2.28711 | 5.08E-08 | miR-30a-3p | -1.87908 | 2.22E-07 |
| miR-22-3p | -2.32179 | 5.25E-08 | miR-3084a-3p | 3.156207 | 2.25E-07 |
| miR-29c-5p | -2.69691 | 5.57E-08 | miR-200a-3p | 3.099923 | 2.30E-07 |
| miR-10b-5p | -2.23485 | 7.00E-08 | miR-365-3p | -2.00236 | 3.26E-07 |

DE miRNAs in EVs-treated rMCs

| Symbol | FC | Adj p value | Symbol | FC | Adj p value |
| --- | --- | --- | --- | --- | --- |
| miR-409 | 2.5642 | 2.40E-07 | miR-181d-5p | -1.90533 | 4.68E-07 |
| miR-134-5p | 2.64611 | 2.48E-07 | miR-24-3p | 3.106189 | 4.68E-07 |
| miR-142-5p | 1.87908 | 2.48E-07 | miR-3573-3p | -2.26116 | 4.86E-07 |
| miR-21-3p | 1.90453 | 2.52E-07 | miR-222-3p | -2.15105 | 5.00E-07 |
| miR-212-3p | 3.099923 | 2.72E-07 | miR-1843b-3p | -2.28587 | 5.02E-07 |
| miR-219a | 3.156207 | 2.73E-07 | miR-6328 | -2.60149 | 5.08E-07 |
| miR-298-5p | 1.60224 | 2.80E-07 | miR-409a-3p | -2.26097 | 5.11E-07 |
| miR-3546 | 2.40326 | 2.99E-07 | miR-181c-5p | -1.91223 | 5.30E-07 |
| miR-455 | 2.257864 | 3.24E-07 | miR-3473 | -1.77101 | 5.30E-07 |
| miR-483-5p | 2.69623 | 3.26E-07 | miR-126a-3p | -1.75378 | 5.38E-07 |
| miR-511-3p | 2.00236 | 3.26E-07 | miR-29a-3p | 2.129895 | 5.55E-07 |
| miR-378-5p | 3.391757 | 3.39E-07 | miR-27a-3p | -2.96069 | 5.59E-07 |
| miR-202-3p | 2.267042 | 3.45E-07 | miR-335 | 4.025019 | 5.67E-07 |
| miR-7a-1-3p | 2.05362 | 3.57E-07 | miR-141-3p | -2.64431 | 5.87E-07 |
| miR-3584 | 2.13705 | 3.59E-07 | miR-503-5p | -2.1174 | 5.97E-07 |
| miR-328a-5p | 3.39317 | 3.71E-07 | miR-191a-3p | -3.91312 | 5.97E-07 |
| miR-193a-3p | 2.51123 | 3.74E-07 | miR-3102 | 1.926622 | 6.11E-07 |
| miR-497-5p | 2.92678 | 3.82E-07 | miR-702-3p | 2.228748 | 6.12E-07 |
| miR-92b-3p | 1.99848 | 3.96E-07 | miR-582-3p | -1.95872 | 6.15E-07 |
| miR-322-3p | 2.02084 | 4.05E-07 | miR-23a-3p | -2.0728 | 6.15E-07 |
| miR-340-3p | -2.06628 | 4.15E-07 | miR-10a-3p | -2.1336 | 6.22E-07 |
| miR-320-3p | 2.898295 | 4.19E-07 | miR-1839-5p | -1.46868 | 6.29E-07 |
| miR-3544 | -4.47074 | 4.24E-07 | miR-30c-5p | -3.60597 | 6.29E-07 |
| miR-194-3p | 4.12477 | 4.24E-07 | miR-200b-5p | -1.68178 | 6.63E-07 |
| miR-375-3p | -1.97507 | 4.54E-07 | miR-193b-3p | 3.26E-07 | 6.67E-07 |
